# *In Vitro* Activity of Delafloxacin and Microbiological Response against Fluoroquinolone-Susceptible and Nonsusceptible Staphylococcus aureus Isolates from Two Phase 3 Studies of Acute Bacterial Skin and Skin Structure Infections

**DOI:** 10.1128/AAC.00772-17

**Published:** 2017-08-24

**Authors:** S. McCurdy, L. Lawrence, M. Quintas, L. Woosley, R. Flamm, C. Tseng, S. Cammarata

**Affiliations:** a Melinta Therapeutics, New Haven, Connecticut, USA; b JMI Laboratories, North Liberty, Iowa, USA; c H20 Clinical, Hunt Valley, Maryland, USA

**Keywords:** ABSSSI, fluoroquinolone, Staphylococcus aureus, delafloxacin, susceptibility

## Abstract

Delafloxacin is an investigational anionic fluoroquinolone antibiotic with broad-spectrum *in vitro* activity, including activity against Gram-positive organisms, Gram-negative organisms, atypical organisms, and anaerobes. The *in vitro* activity of delafloxacin and the percent microbiological response in subjects infected with fluoroquinolone-susceptible and nonsusceptible Staphylococcus aureus isolates were determined from two global phase 3 studies of delafloxacin versus vancomycin plus aztreonam in patients with acute bacterial skin and skin structure infections (ABSSSI). Patients from 23 countries, predominately the United States but also Europe, South America, and Asia, were enrolled. The microbiological intent-to-treat (MITT) population included 1,042 patients from which 685 S. aureus isolates were submitted for identification and susceptibility testing per CLSI guidelines at the central laboratory (JMI Laboratories, North Liberty, IA). The comparator fluoroquinolone antibiotics included levofloxacin and ciprofloxacin. Nonsusceptibility to these antibiotics was determined using CLSI breakpoints. S. aureus isolates were 33.7% levofloxacin nonsusceptible (LVX-NS). The delafloxacin MIC_90_ values against levofloxacin-nonsusceptible S. aureus, methicillin-resistant S. aureus (MRSA), and methicillin-susceptible S. aureus isolates were all 0.25 μg/ml. Delafloxacin demonstrated high rates of microbiological response against LVX-NS isolates as well as isolates with documented mutations in the quinolone resistance-determining region (QRDR). S. aureus was eradicated or presumed eradicated in 98.4% (245/249) of delafloxacin-treated patients. Similar eradication rates were observed for delafloxacin-treated subjects with levofloxacin-nonsusceptible S. aureus isolates (80/81; 98.8%) and MRSA isolates (70/71; 98.6%). Microbiological response rates of 98.6% were observed with delafloxacin-treated subjects with S. aureus isolates with the S84L mutation in *gyrA* and the S80Y mutation in *parC*, the most commonly observed mutations in global phase 3 studies. The data suggest that delafloxacin could be a good option for the treatment of infections caused by S. aureus isolates causing ABSSSI, including MRSA isolates, where high rates of ciprofloxacin and levofloxacin nonsusceptibility are observed. (The phase 3 studies described in this paper have been registered at ClinicalTrials.gov under identifiers NCT01984684 and NCT01811732.)

## INTRODUCTION

Acute bacterial skin and skin structure infections (ABSSSI) are among the most common human bacterial infections (1, 2). Methicillin-resistant Staphylococcus aureus (MRSA) is a frequent cause of ABSSSI in both community and hospital settings, particularly in the United States. In addition to MRSA, bacteria commonly isolated from patients with ABSSSI include streptococci, other staphylococcal species, and Gram-negative bacteria, such as Enterobacteriaceae (3). Chronic infections, especially in patients previously treated with antibiotics, are likely to be polymicrobial, with Gram-negative and obligate anaerobic pathogens being found alongside Gram-positive organisms. Such infections with both Gram-positive and Gram-negative pathogens often require broad-spectrum antibiotic treatment (4–6).

Delafloxacin is a novel investigational anionic fluoroquinolone antibiotic with broad-spectrum *in vitro* activity, including activity against Gram-positive organisms, Gram-negative organisms, anaerobes, and atypical respiratory tract pathogens (i.e., Legionella, Chlamydia, and Mycoplasma) (7–10). Delafloxacin can be administered as an intravenous (i.v.) infusion or orally as a tablet for the treatment of ABSSSI. Two global phase 3 studies of delafloxacin versus vancomycin plus aztreonam in ABSSSI have been completed (ClinicalTrials.gov registration no. NCT01984684 and NCT01811732). Delafloxacin demonstrated noninferiority to vancomycin plus aztreonam using the primary endpoint of an objective response at 48 to 72 h (a ≥20% reduction of ABSSSI lesion spread in the area of erythema, as determined by digital planimetry) (J. Pullman, J. Gardovskis, B. Farley for the PROCEED Study Group, E. Sun, M. Quintas, L. Lawrence, R. Ling, and S. Cammarata, submitted for publication).

Delafloxacin is more active *in vitro* than levofloxacin against most Gram-positive pathogens. Notably, the MIC_50_ of delafloxacin for MRSA strains is at least 64-fold lower than that of levofloxacin and ciprofloxacin (7). The increased potency of delafloxacin relative to that of other fluoroquinolones against Gram-positive bacteria and the enhanced activity of delafloxacin at acidic pH may be influenced by its structure-activity relationship profile. The collaboration between a large N-1 substitution and a weakly polar group at C-8 may influence the potency against quinolone-resistant Gram-positive bacteria, a phenotype common among MRSA isolates. The basicity at C-7 may lead to increased potency at acidic pH (11). Delafloxacin is equally effective at stabilizing cleavable complexes by binding either gyrase or topoisomerase IV in both S. aureus and Escherichia coli and, as such, is considered a dual-targeting fluoroquinolone (11–13). Delafloxacin has also demonstrated a low propensity for resistance development in MRSA strains, with resistance frequency rates ranging from 10^−9^ to 10^−11^ (13).

Due to delafloxacin's enhanced potency against MRSA isolates, it was of interest to further investigate the *in vitro* activity of delafloxacin and the microbiological response in phase 3 clinical trial subjects infected with fluoroquinolone-nonsusceptible S. aureus isolates. Further, microbiological responses were examined for clinical trial S. aureus isolates characterized for mutations in the quinolone resistance-determining region (QRDR).

## RESULTS

### Study populations.

The microbiological intent-to-treat (MITT) population for the two global phase 3 studies consisted of 1,042 subjects (*n* = 518 subjects in the delafloxacin arm; *n* = 524 subjects in the vancomycin plus aztreonam arm), from which 685 S. aureus isolates were submitted for identification and susceptibility testing. The population microbiologically evaluable (ME) at follow-up (MEFUI) for the two global phase 3 studies consisted of 806 subjects (*n* = 410 subjects in the delafloxacin arm; *n* = 396 subjects in the vancomycin plus aztreonam arm). In the MITT and MEFUI populations across the 2 studies, 65.4% (339/518) and 65.9% (270/410) of the delafloxacin-treated subjects, respectively, had a monomicrobial Gram-positive bacterial infection. In the MITT and MEFUI populations, in the pooled delafloxacin arm, 3.3% (17/518) and 3.9% (16/410), respectively, had monomicrobial Gram-negative bacterial infections, 14.1% (73/518) and 13.2% (54/410), respectively, had polymicrobial mixed Gram-negative and -positive bacterial infections, and 5.4% (28/518) and 4.6% (19/410), respectively, had mixed aerobic and anaerobic bacterial infections.

### Antimicrobial susceptibility results.

Table 1 summarizes the delafloxacin activity by MIC against the baseline S. aureus isolates recovered the ABSSSI primary infection site or culture of blood of all subjects in the MITT analysis population by geographic region (United States, Europe, or overall [including South America and Asia]). Of the S. aureus isolates, 33.7% were levofloxacin nonsusceptible, whereas 66.0% of the MRSA isolates were levofloxacin nonsusceptible. Delafloxacin demonstrated potent activity against both S. aureus and MRSA isolates, with MIC_50/90_ values being 0.008/0.25 μg/ml and 0.12/0.25 μg/ml, respectively. In comparison, levofloxacin MIC_50/90_ values were 0.25/4 μg/ml and 4/8 μg/ml, respectively. The percentage of isolates that were levofloxacin nonsusceptible was lower among the S. aureus isolates, including both MRSA and methicillin-susceptible S. aureus (MSSA) isolates, from subjects in Europe than subjects in the United States. Table 2 summarizes the activity of delafloxacin against the levofloxacin-nonsusceptible isolates. The delafloxacin MIC_90_ values against levofloxacin-nonsusceptible S. aureus, MRSA, and MSSA isolates were all 0.25 μg/ml.

**TABLE 1:** Summary of delafloxacin activity by MIC against baseline S. aureus isolates from an ABSSSI site or blood overall and by geographic region^*a*^

Organism	United States (*n* = 717)					Europe (*n* = 283)					Overall (*n* = 1,042)				
	No.	MIC (μg/ml)			% LVX-NS	No.	MIC (μg/ml)				No.	MIC (μg/ml)			% LVX-NS
		Range	50%	90%			Range	50%	90%	% LVX-NS		Range	50%	90%	
S. aureus	511	0.002–4	0.008	0.25	44.4	145	0.002–0.5	0.004	0.008	2.8	685	0.002–4	0.008	0.25	33.7
MRSA	281	0.004–4	0.12	0.25	68.0	7	0.004–0.5			42.9	294	0.002–4	0.12	0.25	66.0
MSSA	234	0.002–0.5	0.008	0.12	15.8	138	0.002–0.12	0.004	0.008	0.7	395	0.002–0.5	0.008	0.03	9.6

a Results are from pooled data for the delafloxacin and comparator treatment arms for the MITT population. MRSA, methicillin-resistant Staphylococcus aureus; MSSA, methicillin-susceptible Staphylococcus aureus; *n*, total number of patients; No., total number of MIC values from isolates cultured at the baseline from specimens from the primary infection site or blood; LVX-NS, levofloxacin nonsusceptible. Overall includes subjects from Asia, Latin America, as well as North America and Europe.

**TABLE 2:** Summary of delafloxacin activity by MIC against baseline S. aureus from an ABSSSI site or blood by levofloxacin susceptibility^*a*^

Organism	Levofloxacin-susceptible isolates				Levofloxacin-nonsusceptible isolates			
	No.	Delafloxacin MIC (μg/ml)			No.	Delafloxacin MIC (μg/ml)		
		50%	90%	Range		50%	90%	Range
S. aureus	455	0.008	0.008	0.002–0.12	232	0.25	0.25	0.004–4
MRSA	101	0.008	0.008	0.002–0.12	195	0.25	0.25	0.004–4
MSSA	358	0.008	0.008	0.002–0.12	39	0.12	0.25	0.004–0.5

a Results are from pooled data for the delafloxacin and comparator treatment arms for the MITT population. MRSA, methicillin-resistant Staphylococcus aureus; MSSA, methicillin-susceptible Staphylococcus aureus; No., number of available MIC values from isolates cultured at the baseline from the primary infection site or blood.

### Efficacy analysis of delafloxacin.

Table 3 summarizes the microbiological response at follow-up for subjects in the MEFUI population with S. aureus isolates from ABSSSI infection sites or blood cultures by the baseline delafloxacin MIC and levofloxacin susceptibility and nonsusceptibility. Delafloxacin demonstrated high rates of microbiological response against levofloxacin-nonsusceptible isolates. On the basis of the results for the ME population (pooled data, delafloxacin treatment arm), levofloxacin-susceptible and nonsusceptible S. aureus isolates were eradicated or presumed eradicated in 98.4% (245/249) of delafloxacin-treated patients (Table 3). Similar eradication rates were observed for delafloxacin-treated subjects with levofloxacin-nonsusceptible S. aureus isolates (80/81; 98.8%) and MRSA isolates (70/71; 98.6%) (Table 3). The microbiological response for one subject with an isolate with a delafloxacin MIC value of 4 μg/ml was presumed eradicated (Table 3). In addition, high rates of response were observed for subjects with monomicrobial S. aureus, MRSA, and MSSA infections (Table 4).

**TABLE 3:** Microbiological response at follow-up for subjects with S. aureus isolates from primary infection site or blood cultures by levofloxacin susceptibility and nonsusceptibility by delafloxacin MIC^*a*^

Organism	Baseline delafloxacin MIC (μg/ml)	*N*1	No. (%) of subjects with:	
			Eradicated/presumed eradicated infection	Persisted/presumed persisted infection
Levofloxacin-susceptible S. aureus			165	3
	0.002	15	15 (100.0)	0
	0.004	44	44 (100.0)	0
	0.008	101	98 (97.0)	3 (3.0)
	0.015	7	7 (100.0)	0
	0.06	1	1 (100.0)	0
Levofloxacin-nonsusceptible S. aureus			80	1
	0.03	3	3 (100.0)	0
	0.12	38	38 (100.0)	0
	0.25	36	35 (97.2)	1 (2.8)
	0.5	3	3 (100.0)	0
	4	1	1 (100.0)	0
Levofloxacin-susceptible MRSA			36	1
	0.004	3	3 (100.0)	0
	0.008	30	29 (96.7)	1 (3.3)
	0.015	3	3 (100.0)	0
	0.06	1	1 (100.0)	0
Levofloxacin-nonsusceptible MRSA			70	1
	0.12	32	32 (100.0)	0
	0.25	36	35 (97.2)	1 (2.8)
	0.5	2	2 (100.0)	0
	4	1	1 (100.0)	0
Levofloxacin-susceptible MSSA			130	2
	0.002	15	15 (100.0)	0
	0.004	41	41 (100.0)	0
	0.008	72	70 (97.2)	2 (2.8)
	0.015	4	4 (100.0)	0
Levofloxacin-nonsusceptible MSSA			10	0
	0.03	3	3 (100.0)	0
	0.12	6	6 (100.0)	0
	0.5	1	1 (100.0)	0

a Results are from pooled data for the MEFUI population. Percentages were calculated as 100 × (*n*/*N*1), where *n* is the number of subjects and *N*1 is the number of subjects for each MIC value. If multiple MIC values were reported per subject per pathogen, the highest value was used. MRSA, methicillin-resistant Staphylococcus aureus; MSSA, methicillin-susceptible Staphylococcus aureus.

**TABLE 4:** Microbiological response at follow-up for subjects with monomicrobial or polymicrobial Gram-positive bacterial infections and polymicrobial Gram-positive and Gram-negative bacterial infections^*a*^

Infection type and baseline target pathogen	No. (%) of subjects with eradicated or presumed eradicated infection/total no. evaluated (%)
Monomicrobial	
Staphylococcus aureus	178/181 (98.3)
MRSA	87/88 (98.9)
MSSA	93/95 (97.9)
Polymicrobial Gram-positive bacteria	
Staphylococcus aureus	40/41 (97.6)
MRSA	14/15 (93.3)
MSSA	26/26 (100)
Polymicrobial Gram-positive and Gram-negative bacteria	
Staphylococcus aureus	26/26 (100)
MRSA	5/5 (100)
MSSA	21/21 (100)

a Results are from pooled data for the delafloxacin treatment arm for the MEFUI population. MRSA, methicillin-resistant Staphylococcus aureus; MSSA, methicillin-susceptible Staphylococcus aureus.

### Efficacy analysis of delafloxacin by isolate QRDR genotype.

Table 5 summarizes the microbiological response at follow-up of subjects in the MEFUI population with baseline S. aureus isolates resistant to levofloxacin or ciprofloxacin by mutation in the QRDR. The predominant mutations observed in the S. aureus isolates were S84L in *gyrA* and S80Y in *parC*. The delafloxacin MIC_90_ for the baseline S. aureus isolates with this mutation was 0.25 μg/ml, whereas the MIC_90_ values for levofloxacin and ciprofloxacin were 8 μg/ml and >8 μg/ml, respectively (Table 5). The microbiological response rates to delafloxacin for subjects with S. aureus isolates with this mutation were 98.6% (68/69). The overall microbiological response rate for all S. aureus isolates with documented QRDR mutations was 98.8% (81/82). Data from this study also demonstrated that delafloxacin MIC values did not increase beyond 0.5 μg/ml until at least double mutations in both *gyrA* and *parC* were observed (Tables 5 and 6). One baseline isolate with such a double mutation was found. The delafloxacin MIC value for this isolate was 4 μg/ml, but despite this relatively high delafloxacin MIC value, the microbiological response for the subject with this isolate was presumed eradicated (Tables 5 and 6).

**TABLE 5:** Analysis of QRDR mutations in S. aureus isolates from the delafloxacin arm of the clinical study resistant to levofloxacin and/or ciprofloxacin and the corresponding microbiological response^*a*^

No. of isolates	QRDR mutation profile				No. of patients with an ME microbiological response at follow-up/total no. evaluated (% eradicated^*b*^)	MIC (μg/ml)								
						Delafloxacin			Levofloxacin			Ciprofloxacin		
	*gyrA*	*gyrB*	*parC*	*parE*		Range	50%	90%	Range	50%	90%	Range	50%	90%
1	E88K S84L	WT	E84G S80Y	WT	1/1 (100)	4			>8			>8		
69^*c*^	S84L	WT	S80Y	WT	68/69 (98.6)	0.12–0.25	0.12	0.25	4 to 8	4	8	8 to >8	8	>8
5^*d*^	S84L	WT	S80F	WT	4/5 (80)	0.25–0.5			8 to >8			>8		
3	S84L	WT	S80Y	P451S	3/3 (100)	0.25–0.5			>8			>8		
1	S84L S85P	WT	S80F	D432N	1/1 (100)	0.5			>8			>8		
3	S85P	WT	S80F	WT	3/3 (100)	0.03			2			8		
1^*d*^	S84L	WT	S80F S80Y	WT	0/1 (0)	0.25			8			>8		
1	WT	WT	S80F	WT	1/1 (100)	0.015			1			4		

a Results are from pooled data for the MEFUI population. ME, microbiologically evaluable; MEFUI, microbiologically evaluable at follow-up for the investigator-assessed response; MIC_50_, lowest MIC that inhibits 50% of the strains (≥10 strains) of a single species; MIC_90_, lowest MIC that inhibits 90% of the strains (≥10 strains) of a single species; QRDR, quinolone resistance-determining region; WT, wild type.

b Documented or presumed eradicated.

c For one subject, a follow-up isolate was obtained, and this isolate was unrelated to the baseline isolate, as determined using pulsed-field gel electrophoresis. Only one of these isolates is counted in Table 6.

d For one subject, the subject had one isolate from blood and one from skin with different QRDR mutations with the same MIC value. Both isolates are counted in Table 5 but only one of these isolates is counted in Table 6.

**TABLE 6:** Microbiological response by MIC for S. aureus by single or multiple QRDR mutations^*a*^

Baseline delafloxacin MIC (μg/ml)	No. of isolates tested	No. of isolates without mutations/total no. of isolates at the indicated MIC value	No. of isolates with the indicated mutation/total no. tested		% of isolates (no. producing the indicated result/total no. tested)	
			Single *gyrA* and *gyrB* and/or *parC* and *parE* mutation	Double *gyr*A and *gyrB* and/or *parC* and *parE* mutations	Eradicated^*b*^ (*n* = 244)	Persisted^*c*^ (*n* = 4)
0.002	15	15/15	0/15	0/15	100 (15/15)	0
0.004	44	44/44	0/44	0/44	100 (44/44)	0
0.008	100	100/100	0/100	0/100	97.0 (97/100)	3.0 (3/100)
0.015	7	6/7	1/7	0/7	100 (7/7)	0
0.03	3	0/3	3/3	0/3	100 (3/3)	0
0.06	1	1/1	0/1	0/1	100 (1/1)	0
0.12	38	0/38	38/38	0/38	100 (38/38)	0
0.25	36	0/36	35/36	1/36	97.2 (35/36)	2.8 (1/36)
0.5	3	0/3	2/3	1/3	100 (3/3)	0
4	1	0/0	0/1	1/1	100 (1/1)	0

a Results are from pooled data for the MEFUI population. Percentages were calculated as 100 × (*n*/*N*1), where *n* is the number of subjects and *N*1 is the number of subjects for each MIC value. If multiple MIC values were reported per subject per pathogen, the highest value was used.

b Documented or presumed eradicated.

c Documented or presumed persisted.

## DISCUSSION

Currently, fluoroquinolone antimicrobial agents, such as levofloxacin or ciprofloxacin, are not utilized as antimicrobial therapy for the treatment of skin and soft tissue infections caused by MRSA (4). This is due, in part, to the high preponderance of fluoroquinolone resistance among these isolates. In a surveillance study of organisms from patients with documented skin and soft tissue infections in U.S. and European medical centers (2010 to 2013), the rates of levofloxacin nonsusceptibility for MRSA isolates were 58.8% and 84.9%, respectively (14). In a 2014 study that examined MRSA isolates from all infection types, the rate of levofloxacin nonsusceptibility was 68.9% (7). These data underscore the need for new treatment options for infections caused by fluoroquinolone-resistant organisms. Delafloxacin is a novel anionic fluoroquinolone antibiotic with broad-spectrum *in vitro* activity and increased potency against Gram-positive isolates, including MRSA, and has been studied in two phase 3 trials for the treatment of ABSSSI. In these global phase 3 clinical trials, delafloxacin-treated subjects demonstrated high rates of microbial eradication, including the eradication of S. aureus isolates that were considered levofloxacin nonsusceptible.

In these studies, the overall rate of levofloxacin nonsusceptibility for MRSA isolates was 66%, with higher nonsusceptibility being seen in the United States (68%) than in Europe (42.9%). The lower percentage of levofloxacin nonsusceptibility may be due to the smaller number of MRSA isolates recovered from subjects from Europe (4.8% of S. aureus isolates). Previous surveillance studies have documented a lower rate of MRSA in European nations than in the United States but a higher rate of levofloxacin nonsusceptibility (14, 15). High rates of eradication of S. aureus, MRSA, and MSSA isolates, including isolates that were levofloxacin nonsusceptible, were observed in delafloxacin-treated subjects. These high eradication rates also extended to subjects with monomicrobial S. aureus, MRSA, and MSSA infections.

When the microbiological response was determined for isolates in which QRDR mutations were identified, similarly high eradication rates were observed. Notably, microbiological response rates of 98.6% were observed for subjects with S. aureus isolates with the S84L mutation in *gyrA* and the S80Y mutation in *parC*, the most commonly observed mutations in global phase 3 studies. The delafloxacin MIC_50/90_ for the S. aureus isolates with the S84L mutation in *gyrA* and the S80Y mutation in *parC* was similar to that for the levofloxacin-nonsusceptible group of organisms, and the MIC_90_ for S. aureus isolates with the S84L mutation in *gyrA* and the S80Y mutation in *parC* was higher than that for levofloxacin-susceptible isolates. These data suggest that whereas the activity of delafloxacin is affected by target modifications/mutations, similar to the case for other fluoroquinolone antibiotics, the slight elevation in MIC values observed for some isolates was not impactful, as illustrated by the high level of microbial eradication observed. In addition, delafloxacin MIC values did not increase beyond 0.5 μg/ml until at least double mutations in both *gyrA* and *parC* were observed. One baseline isolate with such a double mutation was found, and the delafloxacin MIC value for this isolate was 4 μg/ml. Despite this relatively high delafloxacin MIC value, the microbiological response for the subject with this isolate was presumed eradicated. It is difficult to extrapolate these findings on the basis of the outcome with this single isolate; more data on outcomes in patients with isolates with elevated baseline MIC values are needed. Previous studies have demonstrated that the pH of skin and abscesses can range from 4.2 to 5.9 and 6.2 to 7.3, respectively (16, 17). It is possible that the enhanced potency of delafloxacin at acidic pH could have played a role in the presumed eradication of this particular pathogen, but this would be difficult to definitively prove. Finally, no emergence of resistance during therapy was observed for any isolates in these studies. In conclusion, these data suggest that delafloxacin may be considered a treatment option for S. aureus isolates, including MRSA isolates, causing ABSSSI where high rates of ciprofloxacin and levofloxacin nonsusceptibility are observed.

## MATERIALS AND METHODS

### Study design and efficacy endpoints.

Delafloxacin was studied in two phase 3, multicenter, stratified, randomized, double-blind trials (studies 302 and 303) designed using the guidelines of FDA (18) and the European Medicines Agency (19). A total of 1,510 subjects from sites in 23 countries, including sites in the United States (62.3%), Europe (30.2%), South America (6.0%), and Asia (1.5%), were enrolled. The enrollment period spanned from April 2013 to January 2016. Patients with ABSSSI were randomly assigned in a 1:1 ratio to receive either delafloxacin at 300 mg i.v. or 450 mg orally every 12 h or vancomycin at 15 mg/kg of body weight i.v. with aztreonam. All vancomycin-treated patients received aztreonam for empirical coverage of Gram-negative bacteria, and this was discontinued once Gram-negative bacterial infections were ruled out. Study 302 used delafloxacin at 300 mg every 12 h i.v. only, while study 303 used delafloxacin at 300 mg every 12 h i.v. for 3 days with a mandatory blind switch to delafloxacin at 450 mg orally every 12 h thereafter. The total treatment duration was 5 to 14 days, according to the investigators' discretion. For study 302, the median duration of treatment with delafloxacin was 5 days, and the median duration of treatment with vancomycin-aztreonam was 5.5 days; for study 303, the median duration of treatment with delafloxacin or vancomycin-aztreonam was 6.5 days. In order to be enrolled, patients had to meet entry criteria and have wounds, burns, major abscesses, or cellulitis with an area of erythema of ≥75 cm^2^ and at least 2 systemic signs of infection (Pullman et al., unpublished). Patients were evaluated at screening, daily while they were on therapy, at a follow-up visit (FU; day 14 ± 1), and at a late follow-up visit (LFU; days 21 to 28). Efficacy was evaluated through assessments of signs and symptoms of infection, measurement of lesion size by digital planimetry, and culture and susceptibility testing of bacterial isolates. An independent ethics committee or institutional review board at each site approved the study protocol, and the trial was conducted in accordance with the Declaration of Helsinki and International Conference on Harmonisation Good Clinical Practice. All patients provided written informed consent.

### Analysis sets.

Analysis of various sets of data was used to evaluate the clinical response and the microbiological response. Details of the data sets used for analysis of the microbiological response are included here. The intent-to-treat (ITT) analysis data set included all subjects who were randomly assigned to a treatment. The microbiological intent-to-treat (MITT) analysis data set included all subjects in the ITT analysis data set who had a baseline bacterial pathogen identified by the sponsor that was known to cause ABSSSI. The microbiologically evaluable (ME) analysis data set included all subjects in the MITT analysis data set who also met the criteria for the corresponding clinically evaluable (CE) analysis data set for the objective or investigator-assessed response. All subjects in the microbiological analysis data sets were analyzed according to their assigned treatment.

### Microbiology outcomes.

The microbiological responses of patients in the ME and MITT analysis data sets were based on the results of baseline and postbaseline cultures (follow-up [MEFUI] and late follow-up [MEFLI]) and susceptibility testing, together with the clinical response assigned by the investigator.

The definitions of documented eradicated, presumed eradicated, and documented persisted were as follows. For documented eradicated, the baseline pathogen was absent in cultures of specimens from the original site of infection at the postbaseline visit. The investigator-assessed response was not considered a determining factor for this microbiological response definition. For presumed eradicated, there was no material available for culture or no culture was done and the patient had an investigator-assessed response of success (cure or improved with total or near resolution of signs and symptoms and no need for further antibiotic treatment). For documented persisted, the baseline pathogen was present in cultures of specimens from the original site of infection at the visit. The investigator-assessed response was not considered a determining factor for this microbiological response definition. For presumed persisted, no material was available for culture or no culture was done and the patient has an investigator-assessed response of failure.

### Microbiology methods. (i) Susceptibility testing.

Isolates were submitted to the central laboratory (JMI Laboratories, North Liberty, IA) for identification and susceptibility testing per CLSI guidelines (20). The comparator fluoroquinolone antibiotics included levofloxacin and ciprofloxacin. Nonsusceptibility to these antibiotics was determined using CLSI interpretative criteria (21). For analysis tables prepared using subject outcome and isolate microbiological data, fluoroquinolone susceptibility/nonsusceptibility was based upon levofloxacin data only. The designation of S. aureus isolates as MRSA or MSSA was based upon oxacillin susceptibility, determined using CLSI interpretative criteria (21).

### (ii) QRDR analysis.

QRDR analysis was performed on fluoroquinolone (ciprofloxacin or levofloxacin)-resistant Gram-positive isolates by testing at the central laboratory. Molecular characterization of the QRDR was performed by PCR amplification of the DNA gyrase (*gyrA* and *gyrB*) and topoisomerase IV (*parC-grlA* and *parE-grlB*) genes, followed by sequencing of the amplicons. The protein amino acid sequences of selected isolates were compared to those of wild-type S. aureus strain NCTC 8325 (22).
